# Occupational skin disorders in a subset of Nigerian hairdressers

**DOI:** 10.11604/pamj.2018.31.100.16499

**Published:** 2018-10-10

**Authors:** Joseph Archibong, Eshan Henshaw, Adebola Ogunbiyi, Adekunle George

**Affiliations:** 1 Dermatology Unit, Department of Internal Medicine, University of Calabar, Nigeria; 2 Department of Medicine, University College Hospital Ibadan, Nigeria

**Keywords:** Hairdressers, occupational skin disorders, Nigeria, dermatoses, contact dermatitis, fungal infection, hand dermatitis, nail disorders, traumatic injuries, onychomycosis

## Abstract

**Introduction:**

Hairdressing is associated with a wide range of disorders. This is particularly true in the African hairdresser, who is saddled with the responsibility of ‘taming’ the rather difficult-to-manage African hair, and is thus exposed to a wide range of chemical, biological and physical materials in the hair grooming process. We therefore sought to determine the prevalence and pattern of occupational skin disorders among hairdressers in Ibadan, one of the oldest and largest cities in Nigeria.

**Methods:**

This was a cross sectional study of hairdressers conducted in 2013 in Ibadan, Nigeria. Hairdressers and their apprentices were interviewed using a structured questionnaire, following which a thorough physical examination was performed to identify any skin disorder.

**Results:**

A total of 226 hairstylists were recruited. The prevalence of occupational skin disorders in the study was 68.13%. The prevalence of specific skin disorders was 32.74% for nail disorders; 28.75% for traumatic skin disorders; and 2.64% for hand dermatitis.

**Conclusion:**

There is a high prevalence of occupational skin disorders among hairdressers, and this may have personal and public health implications.

## Introduction

The terms ‘hairdresser’, ‘hairstylist’, ‘beautician’ or ‘cosmetologist’ refer to anyone whose occupation is to cut or style hair in order to change or maintain a person´s image. It may involve providing one or more of the following: hair grooming services such as shampooing, cutting, colouring, styling of hair and hair extensions; scalp treatments, facial and limb hair removal; application of makeup, and provision of nail and skincare services. To understand the role of the hairdresser in the daily life of the black African woman requires an appreciation of the nature, and texture of the African hair in comparison to that of other races. While there are similarities in morphology and chemical composition, the African hair is more tightly curled and gets tangled easily due to having a higher number of knots. It is also drier, more brittle, less shiny, and more fragile on account of the uneven distribution of sebum and moisture along the hair shaft. These properties make the hair susceptible to breakage, and more difficult to manage [1-3]. On account of the foregoing, various methods and products are employed in managing the hair, thus the need for trained professionals and the attendant exposure of hairdressers to occupational risks. Occupational disease is simply one caused or worsened by exposure to work related risk factors. In Europe, occupational skin diseases (OSD) account for a large proportion of occupational diseases with occupational hand eczema (OHE) being the most frequently recognized, and one with a high annual cost to the society [4]. In a multi-centered survey in Italy, hairdressing was among the first five occupations responsible for over 60% of total cases of occupational Contact dermatitis [5].

A study in Nigeria revealed needle stick injuries, reactions to hair relaxing creams, cuts, burns, electric shocks and low back pain as occupational diseases among hairdressers, with OHE reported by 5% of respondents [6]. Irritant and allergic contact dermatitis of the hand is said to be the main occupational hazard for hairdressers [7]. Due to the fact that most contact is made with the hands and forearms, these regions are often largely affected, but spread to other parts of the body may also occur [8]. Hairdressers have a high risk of developing contact dermatitis from frequent wet work. The continued unprotected low grade exposure to water, detergent and various hairdressing chemicals affects their quality of life and compliance to work [6]. Skin changes was found in 70% of hairdresser trainees in the course of training, 20% of whom gave up the job on account of contact dermatitis [9]. In a multicenter epidemiological survey in Italy, Sertolli *et al.* [5]. found that 42% of hairdressers compared to 23% of teachers suffered or had been suffering from hands and/or forearms exanthems, 61% as against 15% thought it was work-related. Contact dermatitis often does not lead to hospitalization, and a minor degree may be regarded as a normal hazard of life, however, the physical and psychological impact of occupational contact dermatitis may be considerable and the total economic impact high [10]. It frequently evokes strong negative emotions such as frustration, embarrassment and depression [11]. In the hairdresser's work environment, numerous substances which may cause contact dermatitis on skin exposure include: water, shampoos, detergents, conditioners, dyes, bleaches, permanent wave solutions and glove components. Contributing physical factors include heat, sweating and hair drying [12, 13]. Irritant contact dermatitis occurs when physical and/or chemical damage to the barrier layer of the skin exceeds the skin's ability to repair the damage, while allergic contact dermatitis develops when skin which has been sensitized to a particular substance (allergen) gets exposed to that substance again. Dermatitis in hairdressers is multifactorial, once established it is often difficult to manage despite changing jobs and avoiding allergens. Thus, education and prevention is the best and most useful approach [14, 15]. Occupational skin disease impacts negatively on the finances of the individual as well as the community. It also has medical, social and psychological implications and may evolve into a chronic persistent disability [14, 16].

## Methods

This was a cross sectional epidemiological survey involving registered hairdressers/apprentice hairdressers in Ibadan, a city in Western Nigeria. It involved the administration of structured questionnaires to hairdressers and apprentices aged over 18years, who were practicing within the study population and consented to the study. Physical examination was performed by a dermatologist. A multi-stage sampling technique was employed wherein 10 out of the existing 12 registered hairdressing zones were purposively selected. Each zone had about 30 hairdressing salons from which 20 were randomly selected using the ‘Hat and Draw’ method, bringing to a total of 200 salons recruited for the survey. Four hundred questionnaires were to be administered, two in each salon-one to a qualified hairdresser and the other to an apprentice. However, a good number of shops had no apprentices, thus the instruments were eventually administered to 203 qualified hairdressers and 23 apprentices resulting in a total of 226 study subjects.

**Ethical approval**: Ethical approval was requested and obtained from the Institute for Advanced Medical Research and Training of the College of Medicine, University of Ibadan, Nigeria.

**Data collection**: The hairdressers and apprentices were interviewed using a self-administered structured questionnaire. Demographic data such as age, sex, religion and educational status were obtained as well as information on job description, duration on the job, safety precautions at work, presence of skin rash and other disorders since commencement of work. Personal and family history of atopy and nickel sensitivity was sought. Information was obtained to evaluate the presence and effects of occupational, domestic, psychosocial and economic burden of skin disorders. A general physical examination was subsequently performed on all subjects to ascertain the presence of skin disorders. Adequate lighting was provided, and privacy ensured. Data generated was entered and analyzed using the Statistical Package for Social Sciences (SPSS) version 19.0 software (SPSS Inc., Chicago, IL, USA). Simple statistical techniques including mean plus standard deviation, frequency and percentage were employed in reporting results.

## Results

**Socio-demographic data**: Two hundred and twenty six (226) hairdressers/apprentices were recruited for this survey. The mean age was 36.10 ± 8.24 and the highest frequency was among persons aged 30-39 years. Less than 14% had acquired tertiary level education. Table 1 shows the detailed socio-demographic characteristics of the study participants.

**Table 1:** Socio-demographic variables of participants in the study

Variables	Frequency	Percentage (%)
**Age (years)**		
≤ 19	2	0.9
20-29	46	20.4
30-39	96	42.5
40-49	70	31.0
>50	12	5.3
Mean ± SE = 36.10±8.24		
**Marital status**		
Single	17	7.52
Married	201	88.94
Separated	1	0.44
Divorced	4	1.77
Widowed	3	1.33
**Religion**		
Christianity	121	53.54
Islam	105	46.46
**Educational level**		
None	18	8.0
Primary	34	15.0
Secondary	143	63.3
Tertiary	31	13.7

**Prevalence of occupational skin disorders**: One hundred and fifty four subjects had occupational skin disorders (OSD) accounting for a prevalence of 68.13%. The various types of OSD in decreasing order of frequencies were nail disorders, traumatic skin injuries and hand dermatitis. Table 2 describes the result in details.

**Table 2:** Prevalence of groups of occupational skin disorders in the study

Skin disorders	Frequency	Percentage (%)
Traumatic skin injuries	65	28.75
Nail disorders	74	32.74
Hand dermatitis	15	6.64
**Total**	154	68.13

**Pattern of nail disorders**: Chronic paronychia had the highest prevalence among nail disorders accounting for over two third of the observed nail conditions (Table 3).

**Table 3:** Pattern of nail disorders in hairdressers

Nail Disorder	Frequency	Percentage (%)
Chronic paronychia	48	64.86
Onychomychosis	20	27.03
Onychoschisis	4	5.41
Lamellar splitting	2	2.70
**Total**	74	(100.0)

**Figure 1:**
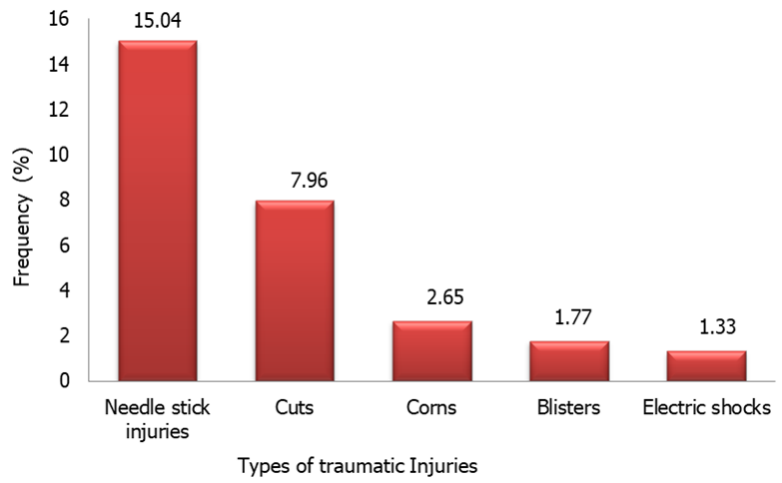
Pattern of traumatic injuries

**Pattern of traumatic injuries**: A total of 65 (28.75%) hairdressers experienced traumatic injuries in the course of their jobs. These consisted of needle stick injuries, cuts from sharps, corns from instruments like scissors, blisters from burns, and electric shock from faulty dryer sockets and hair tongs. Figure 1 shows the relative frequencies of traumatic injuries in hairdressers.

**Prevalence of hand dermatitis**: Six hairdressers reported having observed hand dermatitis prior to the commencement of their various occupations, but while on the job, 15 (6.64%) hairdressers (inclusive of those with hand dermatitis before the commencement of the job) gave a report of hand dermatitis. On physical examination however, hand dermatitis was observed in 12 of 15 hairdressers.

## Discussion

Occupational skin diseases (occupational dermatoses) are those caused or made worse by exposure to work-related hazards [9, 17]. These hazards in the hairdresser's work environment come in chemical (e.g. irritants from hair dyes and relaxers), biological (e.g. fungi from hair/nail infections), or physical (e.g. heat) forms. All the hairdressers in this study were female. This is in keeping with the findings of a skill survey report in the United Kingdom [18] which showed 89% female predominance. The hairdressing profession has traditionally been a female dominated one, as it mostly caters for the haircare needs of females. Males on the other hand predominate in the barbering sector, attending to the hair grooming needs of fellow men. This picture reflects the long held societal norms, which had strict gender-based roles and responsibilities. In recent times however, these demarcating lines are becoming blurred, evidenced by the increased influx of males into the field of hairdressing [19]. In addition, the beauty sector which incorporates skin, nail, and hair care has burgeoned into a huge global financial industry, where persons irrespective of gender have become stakeholders.

Approximately 95% of the hairdressers were women of reproductive age, and close to 90% were already married, howbeit of low socioeconomic status when viewed from their educational background-close to 90% did not attain tertiary level education. They had no formal structured training in hairdressing, mostly acquiring the requisite skill sets via apprenticeship in salons. There are two main pathways to becoming a hairdresser in Nigeria, the first is by registering in a professional training institution which has formal curriculum and practice guidelines, teaches both theoretical and practical aspects of hairdressing, and awards diplomas at the end of the training. There are only a handful of such institutions in the country, they are expensive, and involve months of training. The second route is informal, and involves apprenticeship. Here a trainee is attached to a salon and learns by observing a hairdresser at work. There is no structured training, and no certification. Majority of the subjects belong to the latter category, and are likely to be oblivious to occupational skin diseases in their trade. The implications of these include increased potentials for developing OSD in the absence of requisite pre-job preventive training [20]. In addition, late presentation with subsequent delayed diagnosis of these conditions is more likely among this group, leading to progression and resultant chronicity and/or disability [21]. The high prevalence of OSD among hairdressers is in keeping with studies which found hairdressing to be among occupations with increased risk of occupational contact dermatitis [22, 23]. This is due to repetitive wet works, which is the leading cause of irritant contact dermatitis (ICD) [17]. Infrequent use of gloves exposes hairdressers to frequent contact with water, which causes cumulative sub-toxic contact dermatitis, and is one of the most important predisposing factors for the development of hand dermatitis [24]. Added to the subliminal assault from repetitive wet-works is the corroding effect of irritant chemicals found in hair relaxers, dyes and detergents, which result in skin and nail plate maceration, and eventuate in the higher prevalence of OSD in hairdressers. About 90% of occupational skin diseases are forms of dermatitis [24]. The prevalence of OSD in Studies in Germany and Japan were 55.1% and 49.0% respectively [25, 26] which was slightly lower than what we obtained. While it is logical to ascribe these lower rates to the type of professional training required to become a hairdresser in developed economies as compared to developing ones such as Nigeria where licensure is not a prerequisite, studies among hairdressers and apprentices in the former climes have shown poor knowledge of skin hazards, suboptimal translation of knowledge into practice, and inadequate use of gloves [27, 28]. Diepgenhas also posited that the prevalence of OSD in a number of European studies is largely underestimated [29].

Guo *et al*. [30] obtained a much higher prevalence of 95%, a large proportion of which was hand dermatitis (83%), this condition often has a low prevalence in blacks. Previous studies in Nigeria showed a prevalence of 5% [6] and 14.6% (lifetime prevalence) [31]. While there might be a variety of reasons for this, a known factor which was corroborated in a review by Robinson MK [32] points to the lesser skin reactivity in blacks. Nail disorders accounted for the highest overall prevalence of OSD in the study. More than 90% of the nail disorders were of fungal origin (chronic paronychia and onychomycosis). Nail disorders are infrequently reported in studies among hairdressers in western countries. This is corroborated by Allouni *et al.* [33], who on account of this rarity reported a case of chronic paronychia in a hairdresser, which was a consequence of a hair shaft penetrating beneath the nail fold. A much lower prevalence of 13.7% was obtained among hairdressers in Japan [26]. Studies in Poland showed an increased incidence of fungal, viral and bacterial nail infections among manicurists, and pedicurists, but not among hairdressers [34, 35]. Manicurists have a higher degree of exposure to occupational biological hazards such as bacteria, viruses and fungi, which they may acquire from client's nails and feet [36]. The reason for the low reportage and prevalence among hairdressers may be due to the specialized functions of salon workers in industrialized nations, where there are additional licensing requirements for an individual to provide more than one of the existing salon services viz. manicure, hairdressing, aesthetics etc [37]. This is in contrast to what obtains in salons in our study area, where there is a high level of multitasking, with an individual performing more than one role, oftentimes simultaneously. The pre-existing hand-at-risk (subclinical nail maceration and nail bed inflammation) resulting from prolonged periods of wet works and contacts with numerous chemicals, make hairdressers more predisposed to acquiring infections from clients with tinea or onychomycosis in the course of providing manicure/pedicure services. Poor attitude to the use of hand gloves further compounds the situation.

The high prevalence of traumatic injuries in hairdressers is not unexpected as there is frequent use of sharps such as scissors, razors, and needles in the course of work. A previous survey in this region recorded a higher prevalence of 55% with burns and electrical shocks accounting for a prevalence of 4% and 2% respectively [6]. The least common OSD in our survey was hand dermatitis. Hand dermatitis is the most common dermatoses among hairdressers in numerous surveys among Caucasoid populations [38-40]. The annual prevalence is reported to be 13-22%, although underreporting is common [29, 41]. A point prevalence of 38.6% was obtained in a survey of 60 United Kingdom hairdressing salons [42], while a multicenter survey in France put the prevalence between 12.9-83% [43]. In Nigeria, prevalence rates range from 5%-34.3% [6, 31, 44]. Several reasons can be adduced for the variations in prevalence rates in different populations, these include differences in definitions of OCD, variation in data collection systems (individual reports versus reports and examination of subjects), disparity in subject selection (e.g. qualified hairdressers versus apprentice hairdressers), differing workplace conditions (e.g. work hours, task specificity versus multitasking), investigator proficiency (dermatologists versus non-dermatologists), variations in the type of study outcome reported (point prevalence, annual prevalence, lifetime prevalence etc.), and ethnic differences in skin reactions to irritants [45, 46]. It is highly probable that an important reason for the relatively low prevalence of contact dermatitis in our study is the marked use of potent corticosteroid creams both for skin bleaching, and as self-medication in the treatment of sundry skin disorders. The anti-inflammatory property of corticosteroid may have prevented the development or treated the sub-clinical dermatitis in this group.

## Conclusion

Hairdressers have a high prevalence of occupational skin disorders, many of which have both individual and public health implications if not addressed. The high prevalence of fungal nail disorders can make the hairdresser a source for dissemination of superficial fungal infection amongst clients. The equally high prevalence of cuts and needle stick injuries can also predispose them to both acquire and spread blood borne pathogens such as Hepatitis B and C and the Human Immunodeficiency Virus (HIV). Contact dermatitis can lead to disability, poor quality of life, and eventual job loss. There is a need to create awareness and educate hairdressers on how to prevent and manage occupational skin disorders.

### What is known about this topic

- Hairdressers are at an increased risk of occupational disorders;
- Irritant and contact dermatitis of the hand is the main occupational hazard in hairdressers in developed countries;
- Occupational skin diseases can lead to chronic persistent disability, with medical and psychosocial impact on the affected person.

### What this study adds

- The prevalence and pattern of occupational dermatoses in hairdressers in an African population is now known;
- Nail disorders are the most common occupational dermatoses in Nigerian hairdressers;
- Clients visiting Nigerian hair salons are at risk of acquiring fungal infections, due to the high prevalence of fungal nail infections in hairdressers.

## Competing interests

The authors declare no competing interests.
